# Permittivity Characterization of Conductive and Corrosive LiBr Water Solutions, Method Validation up to 9 GHz Using a Low-Cost SMA Probe

**DOI:** 10.3390/s25030789

**Published:** 2025-01-28

**Authors:** Anne-Laure Perrier, Gregory Houzet, Jonathan Outin, Edouard Rochefeuille, Benoit Stutz, Thierry Lacrevaz

**Affiliations:** 1 LOCIE Laboratory, Université Savoie Mont Blanc, CNRS UMR 5271, Savoie Technolac, 73376 Le Bourget du Lac, France; jonathan.outin@univ-smb.fr (J.O.); benoit.stutz@univ-smb.fr (B.S.); 2 CROMA Laboratory, Université Savoie Mont Blanc, CNRS UMR 5130, Savoie Technolac, 73376 Le Bourget du Lac, France; gregory.houzet@univ-smb.fr (G.H.); edouard.rochefeuille@univ-smb.fr (E.R.); thierry.lacrevaz@univ-smb.fr (T.L.)

**Keywords:** permittivity characterization, microwave measurements, high-conductivity solution, LiBr water solutions

## Abstract

In this article, we present a method for extracting the complex permittivity of high-conductivity solutions up to 9 GHz. Microwave measurements were performed using a low-cost SMA connector, employed as an open-circuit coaxial probe, which was subsequently brought into contact with the liquids under characterization. Compared to state-of-the-art techniques, this method offers the advantage of good accuracy while remaining simple to implement with a low-cost sensor. The affordability of the sensor is crucial because the sensor must operate in a corrosive environment. The use of existing but expensive commercial solutions is prohibitive. Therefore, sensor replacement must be straightforward and inexpensive in case of damage. Two permittivity extraction methods were studied, both relying on a straightforward experimental approach and knowledge of the complex permittivity of reference liquids (deionized water, ethanol, methanol). The technique was initially validated on saline solutions (NaCl) known from the literature before being applied to aqueous lithium bromide (LiBr water) solutions. Eight LiBr water solutions, known to be highly corrosive, were measured for LiBr mass concentrations ranging from 1% to 54% and for conductivities up to 14 S/m. The high conductivity of these solutions brings challenges to extract the real part of the permittivity, which is underestimated by both methods. In contrast, the imaginary part exhibits consistent results with variations strongly correlated to the concentration. Notably, an inversion of the direction of variation was observed for mass concentration in LiBr exceeding 35% aligning with the conductivity curve.

## 1. Introduction

Absorption machines are thermodynamic machines that combine an engine cycle with a heat pump cycle. They are primarily used to extract heat from a cold source (such as buildings during summer at 23 °C, for instance) and reject it to the ambient environment (ambient temperature at 35 °C, for instance) by converting the thermal energy transferred from a hot source (heat from solar thermal collectors at 90 °C) to the ambient environment. The water–lithium–bromide pair (LiBr/H_2_O) remains the best-performing thermochemical pair for chilled water production or air conditioning [1].

In the current energy context, where research is focused on recovering waste energy, the use of such machines is becoming increasingly relevant. Optimizing these machines requires access to detailed information on the temperature and concentration distributions of these LiBr water solutions.

The objective of the present work was to quantify the variation in permittivity of LiBr water solutions as a function of their mass concentration over a frequency range from a few MHz to 9 GHz. Additionally, a permittivity characterization technique was developed to address the challenges brought by the high corrosiveness of LiBr. In this study, we focus only on variations of the LiBr mass concentration at a fixed room temperature of 22 °C. The ultimate goal is to determine whether permittivity variations would be sufficient at a given frequency or over a given frequency range in order to accomplish a LiBr concentration and/or temperature sensor. This sensor will have to be sensitive to LiBr water solutions with LiBr mass concentrations ranging between 35% and 60% and temperatures from 25 °C to 45 °C, corresponding to the operational conditions of absorption machines.

The work presented on LiBr water solutions is not limited to the concentration range used in absorption machines. Indeed, this study was carried out over the entire mass concentration range possible at room temperature, i.e., from 1% to about 54%.

In the radiofrequency field, the six techniques used to characterize permittivity are: the open-ended coaxial probe method, the transmission line method, the free space method, the resonant cavity method, the parallel plate method, or the inductance measurement method.

Although these different techniques are used to characterize liquids, or other research projects to exploit specific circuits such as filters [2], resonant circuits [3,4,5], or waveguide probes [6], the coaxial probe method remains the most widely used technique for characterizing liquids. We employed this technique due to its advantages of being broadband and easy to set up. Several studies have been carried out using this technique, with the main challenge being to calibrate the system in the measurement plane, i.e., at the probe–liquid interface, to account for all error parameters associated with S-parameter measurements. Various teams have worked on calibration using different reference liquids [7], creating their own calibration kits [8], or implementing specific algorithms [9]. The most widely used calibration method remains the Open–Short–Load in the liquid–probe plane, and some commercial products based on the coaxial probe method using this calibration technique are also available [10]. Our measurements were motivated by a very low-cost probe (the aim is to have a disposable probe in case it is damaged by the solution, rather than investing in an expensive commercial probe that will be immersed in a corrosive liquid), full control of the various stages from S-parameter measurement to permittivity extraction (Commercial systems provide permittivity results directly, but do not grant access to S-parameter measurements or the extraction technique used. In the event of incorrect results, it is impossible to determine the cause of the problem) and search for the simplest possible experimental technique (no need to manufacture a specific calibration kit or use electromagnetic simulations). For these reasons, we have chosen to use a low-cost commercial SMA connector (less than EUR 10) as a coaxial probe, as well as two different techniques to characterize our solutions: the method of Wagner et al. [7] based on an Open–Water–Liquid (OWL) calibration and the method of Lacrevaz et al. [11]. The non-use of the short-circuit calibration element in the probe–liquid plane remains an advantage of these two methods. Indeed, this calibration element is experimentally difficult to implement when homemade. For both methods, a calibration step using the calibration kit supplied with the vector network analyzer (VNA) is performed at the probe input. The first method requires at least three reference media to perform the calibration step in the liquid–probe plane while the second one requires two reference media and the probe properties (propagation constant and losses).

To our knowledge, LiBr water solutions have not been characterized in the microwave spectrum to extract their permittivity; we have no reference works to compare our results with. The difficulty is increased by the very high corrosivity of these solutions, which requires the use of specific test cells, safe for the manipulator and composed of suitable materials. The corrosiveness of LiBr excludes the use of certain metals such as copper, which is generally used in the radio-frequency field, and certain polymers. To compare our work with the literature, we have also characterized the permittivity of various sodium chloride (NaCl) water solutions.

In the state of the art, several studies have been done to measure and/or to fit the complex permittivity as a function of frequency using an equation model of pure water [12], various salt solutions such as seawater [13,14,15,16,17,18], pure NaCl water solutions [19,20,21,22,23], or other specific liquids [24,25,26,27]. Many of these studies have demonstrated a strong correlation between the complex permittivity of a liquid and the temperature–concentration couple.

The referenced works are those of Cole–Cole [28], Cole–Davidson [29], or Debye [30]. Over time, various works have improved and refined the parameters for fitting complex permittivity curves as a function of temperature, frequency, or concentration of the various solutions. The theoretical modeling of our permittivity variation as a function of frequency is based on two models described in the literature: one to validate our technique using NaCl water solutions [22], and the other to provide a model for our reference liquids [7] (deionized water, ethanol, and methanol).

To summarize, we present a microwave study (up to 9 GHz) of the extraction of the complex permittivity of corrosive solutions used in the field of absorption machines. This paper is structured in four parts.

After this first introduction section, the second section entitled “Materials and Methods” is split according to the following division: (i) The presentation of theoretical models of a variation of the complex permittivity as a function of the frequency. The first model describes the well-known behavior of our reference liquids already massively illustrated in the literature [7] (ethanol, methanol, deionized water). The second model describes saline solutions (NaCl) and addresses aspects related to ionic electric conductivity. (ii) The experimental device implemented for our measurement campaign is presented. From these microwave measurements, we describe two techniques for extracting permittivity from the measured S parameters. The two techniques presented allow a comparison of the results obtained. The third section provides an analysis of the extracted results allowing us to define the validity domains of the two extraction methods. Finally, the fourth section presents the conclusion of this work.

Because of all these additions, this article is an expanded version of [31], which was presented at the IEEE ICECCME 2024 conference.

## 2. Materials and Methods

### 2.1. Theoretical Models of Complex Permittivity and Preparation of Solutions

The first subsection presents theoretical models of complex permittivity versus frequency of non-conductive liquids. Those liquids will enable the validation and/or calibration of our experimental setup and extraction process. Indeed, their complex permittivities dispersion is well known [7].

The second subsection introduces a liquid that presents an electric conductive part in its complex permittivity. We use NaCl water solutions. Again, those liquids are well known [22]. Theoretical model of permittivity dispersion of NaCl water solutions are used to validate our experimental setup and extraction process applied to conductive liquids.

The last subsection describes the LiBr water solutions we want to characterize. We describe the preparation of these solutions with different mass concentrations. As they present conductivity, we measured those solutions (as well of the NaCl ones) with a Consort K912 conductivity meter at room temperature (22 °C). It should be noted, for a good understanding of the values provided below, that the Consort K912 conductivity meter converts the conductivity measured at a given temperature to the equivalent conductivity at 25 °C (this operation is inherent to the device and cannot be modified).

To our knowledge, there is no study conducted on the complex permittivity of LiBr solutions in the microwave domain. Only [32] presents a study on the real part of the permittivity of LiBr solutions as a function of the concentration, but without frequency dependence.

#### 2.1.1. Reference Liquids: Deionized Water, Ethanol, Methanol

Different liquids with precisely known permittivity can be used as reference and to calibrate the experimental procedures. For our study, we chose deionized water, methanol, and ethanol as references. Their complex relative permittivity can be defined by Equation (1) from the Cole–Cole model [28], extended with the ionic conductivity term, using the different parameters given in Table 1 (from the work of Wagner et al. [7]) and the measured conductivities (using the Consort K912 conductivity meter) given in Table 2:

(1) $$\varepsilon_{r}^{*}\left(\omega ,T\right)=\varepsilon_{\infty }+\frac{\varepsilon_{S}-\varepsilon_{\infty }}{1+{\left(j\omega \tau_{0}\cdot e^{\frac{E_{A}}{RT}}\right)}^{\beta_{cc}(T)}}+\frac{\sigma_{i}}{j\omega \varepsilon_{0}}$$

where *T* is the temperature (in K), $\varepsilon_{r}^{*}$ is the complex relative permittivity of the medium, $\varepsilon_{S}=\varepsilon_{m,S}{\cdot e}^{\alpha_{S}T^{\prime }}$ is the static relative permittivity, $\;\varepsilon_{\infty }=\varepsilon_{m,\infty }{\cdot e}^{\alpha_{\infty }T^{\prime }}$ is the relative permittivity at the high-frequency limit (*T*′ is the temperature in °C, $\varepsilon_{m,S}$, $\varepsilon_{m,\infty }$, $\alpha_{S}$, and $\alpha_{\infty }$ are empirical parameters), $\varepsilon_{0}$ is the vacuum permittivity (in F/m), *ω* is the angular frequency (in rad/s), *σ_i_* is the ionic electric conductivity (in S/m), *R* is the perfect gas constant (8.314 J/(mol K)), *E_A_* is the activation energy (in J/mol), $\tau_{0}$ is the relaxation time (in s), and $\beta_{cc}(T)$ is the stretching exponent or Cole–Cole parameter (assumed to be 1).

**Figure 1:**
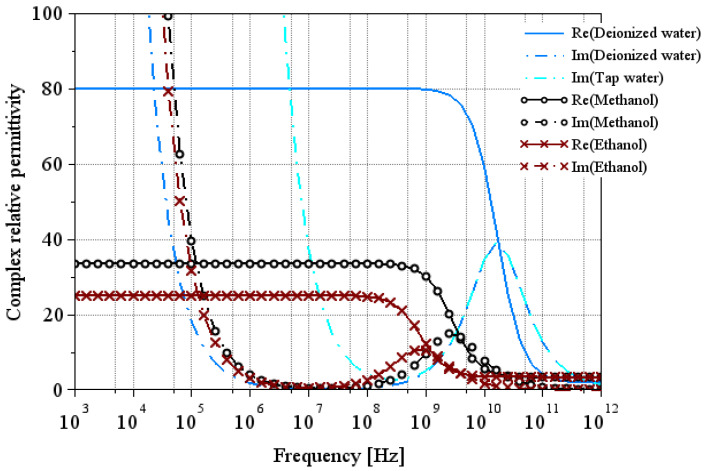
Theoretical model of relative permittivity of reference liquids at 25 °C using Equation (1). The solid line and the dash-dotted line represent the real part and the imaginary part of the relative permittivity, respectively.

Figure 1 shows the real and imaginary parts of the relative permittivity of the reference liquids derived from Equation (1) and using the parameters given in Table 1 and Table 2. This theoretical modeling will later be used to compare our extractions. Note that for liquids with low conductivity (as ethanol, methanol, or deionized water), its impact on the imaginary part of permittivity operates at frequencies below 1 MHz. For tap water, this impact extends up to 10 MHz.

#### 2.1.2. NaCl Water Solutions

As LiBr water solutions have never been characterized in the microwave domain, NaCl water saline solutions known from the literature and having a significant electrical conductivity were used to verify permittivity extraction methods.

For saline solutions, i.e., sodium chloride (NaCl) solutions in water, Equation (1) can be written as follows [21,22]:

(2) $$\varepsilon_{r}^{*}\left(\omega \right)=\varepsilon_{\infty }+\frac{\varepsilon_{S}-\varepsilon_{\infty }}{1+{\left(j\omega \tau \right)}^{1-\alpha }}+\frac{\sigma_{i}}{j\omega \varepsilon_{0}}$$

where the Debye and Cole–Cole parameter $\varepsilon_{S}$, $\varepsilon_{\infty }$, τ, $\sigma_{i}$, and α depend on temperature and saline concentration. The model for concentrations below 1 M as presented in the following equations:

(3) $$\varepsilon_{S}=\varepsilon_{S_{water}}\cdot (1-3.742\cdot 10^{-4}tc+0.034c^{2}-0.178c+1.515\cdot 10^{-4}t-4.929\cdot 10^{-6}t^{2})$$

(4) $$\tau =\tau_{water}\cdot (1.012-5.282\cdot 10^{-3}tc+0.032c^{2}-0.01c-1.724\cdot 10^{-3}t-3.766\cdot 10^{-5}t^{2})$$

(5) $$\alpha =-6.348\cdot 10^{-4}tc-5.1\cdot 10^{-2}c^{2}+9\cdot 10^{-2}c$$

(6) $$\sigma_{i}=0.174tc-1.582c^{2}+5.923c$$

where *t* is the temperature in degrees Celsius, c is the saline concentration in moles per liter (M), $\varepsilon_{S_{water}}$ and $\tau_{water}$ are the values of static relative permittivity $\varepsilon_{S}$ and the relaxation time τ of deionized water, respectively. The parameters $\varepsilon_{S}$, $\varepsilon_{\infty }$, and τ of deionized water are given in article [12] for discrete temperature values. In Table 3, we have recorded the values of these parameters for the closest temperatures to our experimental study, i.e., 20 °C and 25 °C. Equations (1) and (2) are similar for deionized water with α=0 (Equation (2)), $\beta_{cc}=1$ (Equation (1)) and $\tau =\tau_{0}{\cdot e}^{\frac{E_{A}}{RT}}$.

To compare the two models derived from Equations (1) and (2), we recalculated the parameters $\varepsilon_{S}=\varepsilon_{m,S}{\cdot e}^{\alpha_{S}T^{\prime }}$, $\varepsilon_{\infty }=\varepsilon_{m,\infty }{\cdot e}^{\alpha_{\infty }T^{\prime }}$, and $\tau =\tau_{0}{\cdot e}^{\frac{E_{A}}{RT}}$ using the data in Table 1 for deionized water. The results are shown in Table 3.

Five saline solutions with High NaCl concentrations of 0.154 M, 0.25 M, 0.5 M, 1 M, and 2 M were selected in this study to investigate the influence of high conductivity on measurements and permittivity extractions. Parameters of the NaCl water solutions calculated with Equations (3)–(6) are shown in Table 4 for two temperatures close to room temperature. Conductivity measurements (using the Consort K912 conductivity meter, by the Consort company of Belgium) are presented in Table 5. We note that these conductivity measurements, in the right order of magnitude, are slightly underestimated compared to the values expected using Equation (6), given in Table 4. Measurements taken on other liquids with the same measuring instrument are also likely to be slightly affected.

This subsection allows us to plot the theoretical curves of the complex permittivity of a conductive liquid known in the literature [22]. This theoretical modeling will later be used to compare our extractions on a conductive liquid.

#### 2.1.3. Preparation and Static Measurements of LiBr Water Solutions

Water solutions of LiBr at different LiBr mass concentrations were made for characterization. To cover the entire concentration range, two 1 L solutions with a LiBr mass concentration of 55% were made (to limit the heat generated by exothermic dissolution, 873 g of LiBr are dissolved in small quantities of 50 g in deionized water to obtain a 1 L solution). The first 1 L solution was diluted to obtain 500 mL solutions with LiBr mass concentrations of 1%, 5%, 10%, 20%, 30%, and 40%. The second 1 L solution was used to make 500 mL solutions with concentrations of 50% and 55%.

The concentration of these different solutions was then measured with a pycnometer and their conductivities were measured with the Consort K912 conductivity meter at room temperature (22 °C). Mass concentration calculations for LiBr water solutions are based on Boryta’s work [33]. In particular, correlations derived from his work make it possible to relate density values obtained by the pycnometer to mass concentrations. The concentration and conductivity measurements are presented in Table 6. This table also shows the relationship between LiBr mass concentration and concentration in moles/liter, which will be used later for comparisons with a paper in the literature [32]. The differences between the expected and the measured concentrations are certainly due to the precision of the weighing process. Indeed, the two 1 L starting solutions required 17 weighings of approximately 50 g of LiBr each. As a result, one stock solution was probably overdosed with LiBr while the other was underdosed, leading to a propagation of this concentration error during subsequent dilutions. Despite these differences, the error between the desired and actual concentrations is not significant. Thus, the critical factor is precisely determining the concentration of the LiBr water solutions to be characterized, which was achieved using the pycnometer.

**Figure 2:**
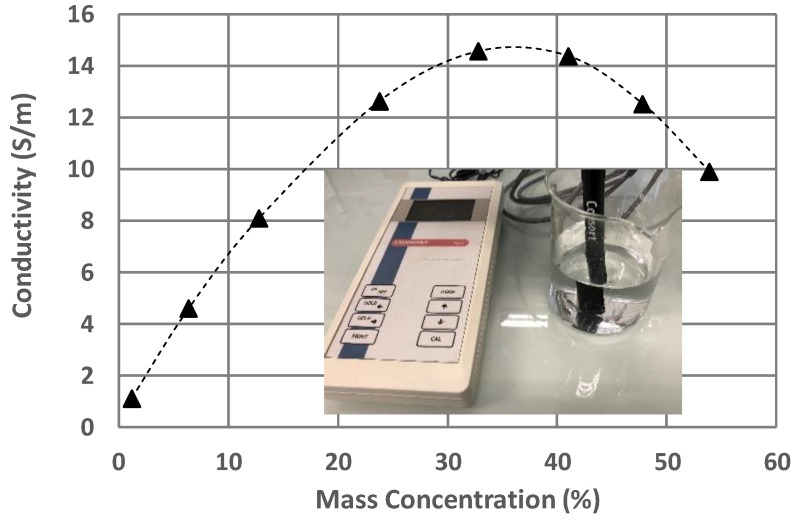
Conductivity of LiBr water solutions as a function of their LiBr mass concentrations. The value of the conductivity is calculated for a temperature of 25 °C by the conductivity meter.

Figure 2 shows the conductivity measurements of the LiBr water solutions at 25 °C as a function of their concentration. The decrease in conductivity observed for high LiBr concentrations (over 35% by mass) has already been observed by different research teams [34,35]. We can assume that the imaginary part of permittivity, which contains conductivity, is affected by this trend.

### 2.2. Experimental Characterization of Complex Permittivity

#### 2.2.1. Experimental Setup

**Figure 3:**
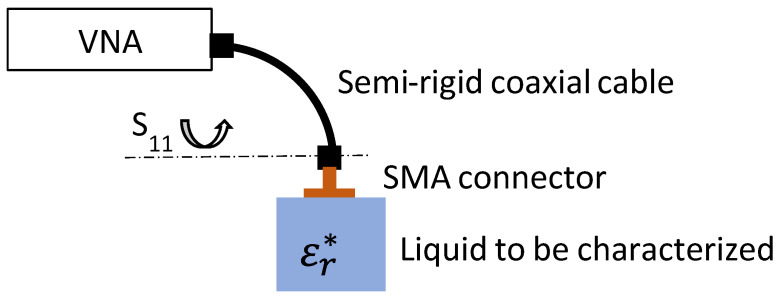
Principle of characterization of LiBr water solutions.

To characterize the different liquids in the microwave domain, we used a coaxial probe in an open circuit immersed in the liquid under test. Microwave measurements of the reflection coefficient (*S*_11_) were performed using a Keysight P9372A 300 kHz–9 GHz vector network analyzer (VNA). Calibration of the measurement system (VNA + semi-rigid coaxial cable) was carried out automatically using an electronic kit (Keysight ecal N7552A commercial device) at the input probe plane. As shown in Figure 3, this reference plane of the measurement (identified by *S*_11_) is located at the output of the male connector of the semi-rigid coaxial cable of 50 Ω characteristic impedance, i.e., at the coaxial probe input.

The coaxial probe, immersed in the liquid under test consists of a female SMA-type connector, typically mounted on the edge of a Würth Elektronik PCB (ref. 60312202114514) and repurposed from its original function. This connector was chosen for its low cost and its resistance to corrosive LiBr water solutions. However, it was modified by removing all of its pins to create a flat surface in contact with the liquid. All measurements were performed following the same calibration; the temperature of the room and, therefore, of the liquids present in the room was measured at 22 °C.

From the measurements of parameter *S*_11_, two complex permittivity extraction methods were implemented and evaluated.

#### 2.2.2. Permittivity Extraction

##### Wagner’s Method [7]

The first method, from the work by Wagner et al. [7], is based on an open-water-liquid (OWL) calibration. The authors demonstrated that for each medium *m*, located at the end of the probe, the complex relative permittivity was related to reflection coefficient *S*_11_ by Equation (7). To follow this method, we used three reference media—air, deionized water, and methanol:

(7) $$S_{11}^{m}c_{1}-c_{2}-\varepsilon_{r,m}^{*}c_{3}=\varepsilon_{r,m}^{*}S_{11}^{m}$$

where $c_{1}$, $c_{2}$, and $c_{3}\;$constitute a matrix *c* allowing us to take into account [7,26]: a capacitance linked to the fringe effect at the end of the probe, the capacitive effect of the medium (air and liquids), as well as the attenuation and the phase shift introduced by the SMA connector. For three reference media, Equation (7) is represented in the matrix form of Equation (8) with *O* for open (air), *W* for water, and *M* for methanol. Note that the S parameter in Equations (7) and (8) are of course frequency dependent, implying that the *c* matrix is also frequency dependent.

(8) $$\left(\begin{array}{ccc} S_{11}^{O} & -1 & \varepsilon_{r,O}\\ S_{11}^{W} & -1 & \varepsilon_{r,W}^{*}\\ S_{11}^{M} & -1 & \varepsilon_{r,M}^{*} \end{array}\right)\left(\begin{array}{c} c_{1}\\ c_{2}\\ c_{3} \end{array}\right)=\left(\begin{array}{c} {-\varepsilon }_{r,O}S_{11}^{O}\\ {-\varepsilon }_{r,W}^{*}S_{11}^{W}\\ -\varepsilon_{r,M}^{*}S_{11}^{M} \end{array}\right)$$

The complex relative permittivity of the three-reference media from Equation (1) is used in Equation (8) to determine the matrix *c* by numerical solution. The constituents of this matrix *c* are then used in Equation (7) to extract the complex relative permittivity of the medium to be characterized (i.e., the different NaCl or LiBr water solutions).

##### Lacrevaz’s Method [11]

**Figure 4:**
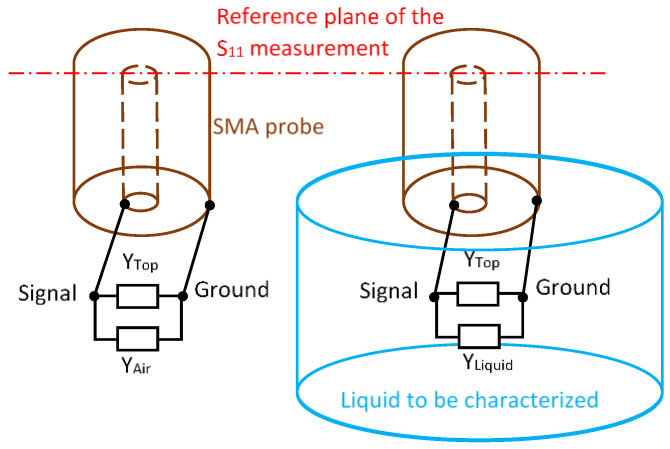
Principle of measurement in air and in liquid associated with electrical models.

The second permittivity extraction method we set up is the one developed by Lacrevaz et al. [11] using two reference media (air and deionized water). This method was developed for a coplanar Ground Signal Ground radiofrequency microprobe on a 1–67 GHz range. In our configuration, the setup of the measurement is described in Figure 4. The SMA probe is charged by two parallel admittances: *Y_Top_* is the admittance including systematic residual errors of calibration and *Y_Air_* and *Y_Liquid_* represent the admittances due, respectively, to the air and the liquid to be characterized.

(9) $$Y_{Air}=j\omega C_{Air}=j\omega \varepsilon_{0}F_{dim}$$

(10) $$Y_{Liquid}=G_{Liquid}+j\omega C_{Liquid}=\omega \varepsilon_{0}{{\varepsilon^{\prime \prime }}_{Liquid}F}_{dim}+j\omega \varepsilon_{0}{{\varepsilon^{\prime }}_{Liquid}F}_{dim}$$

where ${\varepsilon^{\prime }}_{Liquid}$ and ${\varepsilon^{\prime \prime }}_{Liquid}$ are the real and imaginary part of the relative permittivity of the liquid to be characterized and $F_{dim}$ is a factor (in meter) that is related to the geometry and dimensions of the probe tip.

Using two reference media as air and deionized water, a differential admittance ${∆Y}_{Meas}$ is obtained at the probe tip:

(11) $${{∆Y}_{Meas}=Y_{Meas\_water}-Y}_{Meas\_Air}=\omega \varepsilon_{0}{{\varepsilon ’’}_{Water}F}_{dim}+j\omega \varepsilon_{0}F_{dim}{(\varepsilon ’}_{Water}-1)$$

So, we can deduce

(12) $$F_{dim}{=∆Y}_{Meas}/\left[j\omega \varepsilon_{0}{(\varepsilon^{\prime }}_{Water}-j{\varepsilon^{\prime \prime }}_{Water}-1)\right]$$

where ${\varepsilon^{\prime }}_{Water}$ and ${\varepsilon^{\prime \prime }}_{Water}$ are the theoretical real and imaginary part of the relative permittivity of the deionized water (Equation (1)). The dimensioning factor $F_{dim}$ found is then applied to all differential measurements between the liquid to be characterized and air.

To adapt this method to our test configuration, it is necessary to determine the propagation exponent of the SMA connector. Thus, we measured the propagation exponent γ=α+jβ of five identical SMA-female to SMA-female adapters from the same manufacturer and assumed them to have the same electrical characteristics as the SMA probe connector (an average of α ≈ 0.3 Np/m and β = 29.8 rad/m at 1 GHz was determined). The propagation exponent generates the ABCD matrix of the SMA connector. Using the equation for the conversion between S parameters and the ABCD matrix developed by Frickey [36], with port 1 being the VNA reference impedance at 50 ohms and port 2 being the liquid-related load admittance, the complex permittivity performed from the *S*_11_ parameters can be extracted.

## 3. Experimental Results

In this section, we present the complex permittivity extraction results from the measurement of the *S*_11_ parameter by means of the two methods studied (Wagner’s method [7] and Lacrevaz’s method [11]) of the different solutions (reference media, NaCl water solutions, and LiBr water solutions). For improved clarity, the graphs are presented separately, with a focus on the key sections of each graph. This approach highlights the validity domains of each method and emphasizes the relevant results.

**Figure 5:**
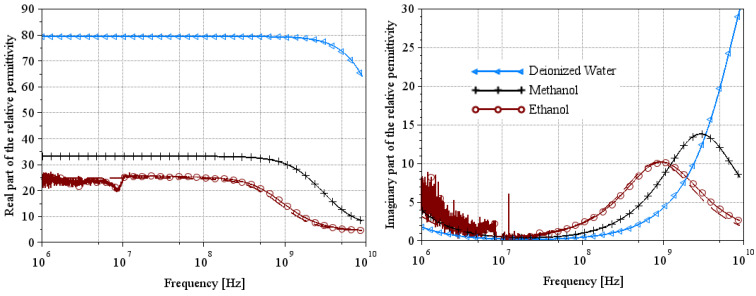
Extraction of the complex relative permittivity of the ethanol using Wagner’s method [7].

**Figure 6:**
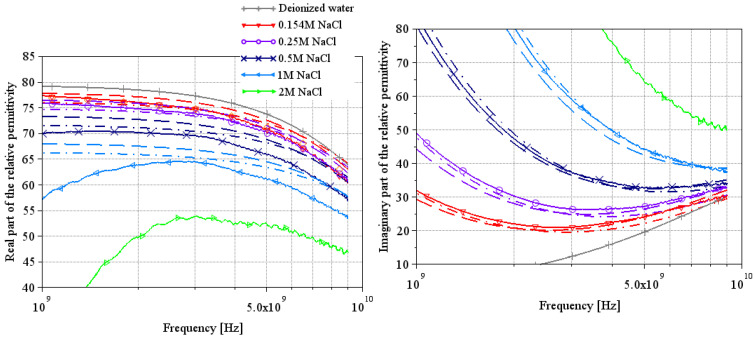
Extraction of the complex relative permittivity of different NaCl water solutions using Wagner’s method [7].

**Figure 7:**
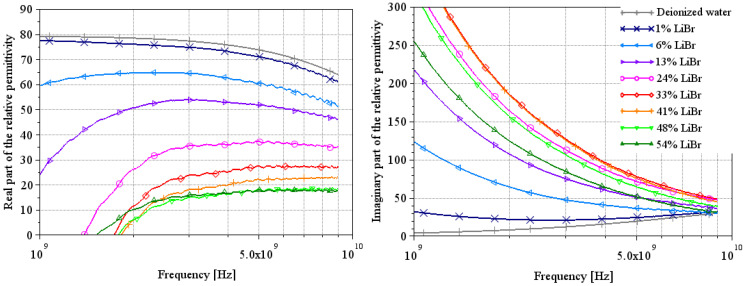
Extraction of the complex relative permittivity of different LiBr water solutions using Wagner’s method [7]. Solid curves are from measurements at a temperature of 22 °C.

**Figure 8:**
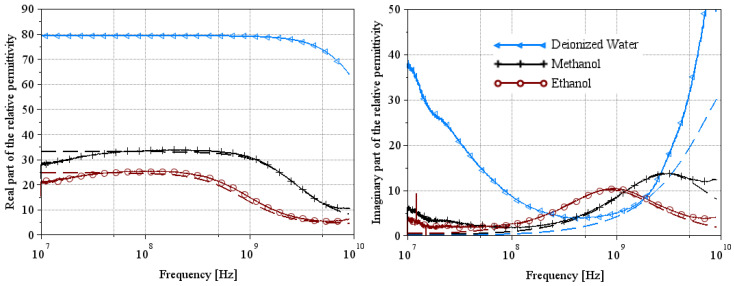
Extraction of the complex relative permittivity of reference liquids (ethanol and methanol) using Lacrevaz’s method [11].

**Figure 9:**
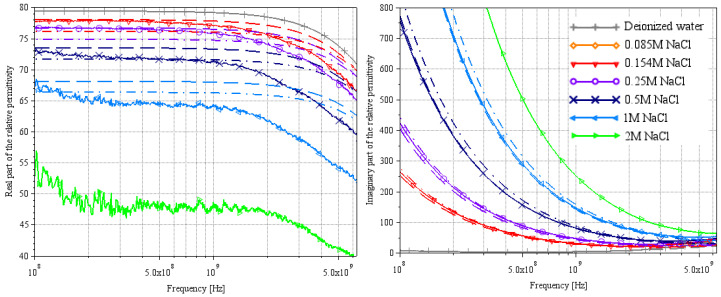
Extraction of the complex relative permittivity of different NaCl water solutions using Lacrevaz’s method [11].

**Figure 10:**
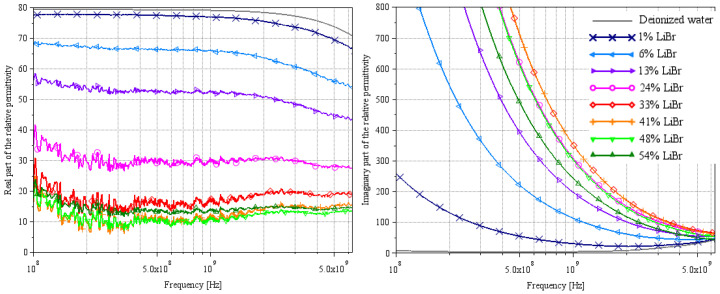
Extraction of the complex relative permittivity of different LiBr/water solutions using Lacrevaz’s method [11].

Figure 5, Figure 6 and Figure 7 and Figure 8, Figure 9 and Figure 10 present, respectively, complex relative permittivity extraction of the different solutions using Wagner’s method [7] and Lacrevaz’s method [11].

### 3.1. Reference Media

Figure 5 and Figure 8 show the complex relative permittivity of the reference media (deionized water, ethanol, and methanol represented in figures by the symbols triangles, crosses and circles, respectively) using the two extraction methods. The solid line represents the measurement results, and the dashed line represents the theoretical results of Equation (1) using parameters of Table 1 and Table 2 for a temperature equal to 22 °C. From these figures, we can see that the permittivity extraction of ethanol for Wagner’s method is in very good agreement with the theoretical curve over the whole frequency range. The deionized water and methanol curves are the theoretical ones used from the extraction method. So, it is clear that the extraction curve matches the theoretical curve perfectly. Note that the artifact located at 10 MHz is undoubtedly a measurement problem linked to the VNA, as it appears on all the measurement curves. Extraction curves for ethanol and methanol using Lacrevaz’s method are very close to theory over a slightly narrower frequency range than for the other extraction method. At low frequencies, below 100 MHz, we believe that the difference between theory and extraction is linked to the artifact that appears at 10 MHz. At high frequencies, we note a slight difference above 6 GHz.

These first extraction results on reference liquids for the two extraction methods make it possible to validate both methods on liquids with very low conductivities.

### 3.2. NaCl Water Solutions

The complex relative permittivities of NaCl water solutions extracted by both methods are shown in Figure 6 and Figure 9. Solid lines are from measurements (T = 22 °C), and the other curves are from theoretical Equation (2) with a dashed line for a temperature of 20 °C and a dash-dotted line for a temperature of 25 °C (using Table 4 parameters).

With Wagner’s method in Figure 6, note that a numerical matrix inversion problem completely disrupts the permittivity extractions, with the real part of the relative permittivity tending towards minus infinity at lower frequencies (not visible in Figure 6: our choice of scale masks the problem to stay focused on the important part of the curve; however, it is clearly visible in Figure 7 for LiBr water solutions). However, the results appear to be consistent with theory above 5 GHz, both for the real and imaginary parts. The extractions of the real part of the permittivity are still underestimated compared to theory; note that the greater the concentration, the greater the error. These results on NaCl water solutions suggest that the results on LiBr water solutions will also be underestimated, but the values around 9 GHz will be plausible.

**Figure 11:**
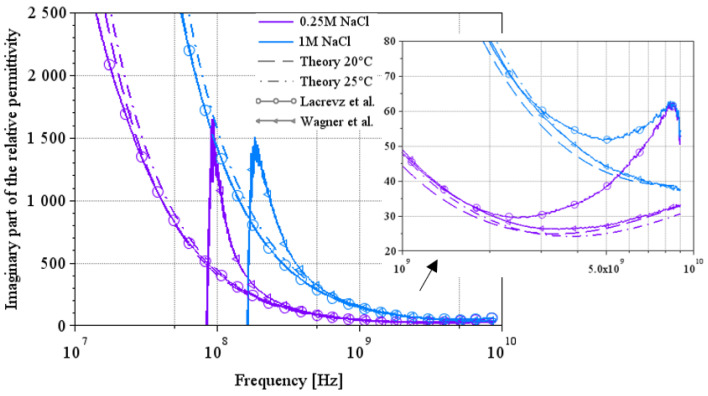
Comparison between the two extraction methods and theoretical results on the imaginary part of the complex permittivity for NaCl water solutions [7,12].

With Lacrevaz’s method in Figure 9, permittivity extractions are consistent with theory, especially in the lower band of the spectrum (between 0.1 and 1 GHz for the real part and below 5 GHz for the imaginary part). The real part of the relative permittivity tends to be underestimated by this method, especially at high frequencies and high concentrations. The imaginary part, on the other hand, is in very good agreement with the theory, except at the end of the spectrum (visible in Figure 11). These results on NaCl water solutions allow us to validate the extraction method for the imaginary part of the permittivity on liquids with high conductivity. However, we must remain cautious about the values extracted for the real part of the permittivity. In Equation (9), conductivity is included in the real part of the admittance $Y_{Liquid}$ (the conductance $G_{Liquid}$). Due to the significantly larger conductance compared to the imaginary part of the admittance ($\omega C_{Liquid}$), extracting the real part of the permittivity becomes very challenging. Even a small error in conductivity can result in a substantial error in the real part of permittivity.

These permittivity extraction results on liquids with theoretical models known from the literature allow us to assume that the results using Lacrevaz’s method for the real part of the permittivity of LiBr water solutions will be highly underestimated. Yet, the results for the imaginary part will be correct up to a few GHz.

### 3.3. LiBr Water Solution

The complex relative permittivities of LiBr water solutions extracted by both methods are shown in Figure 7 and Figure 10 for the most plausible part of the spectrum.

For the real part of permittivity, we remain cautious about the results, but we can say that the values found at 100 MHz by Lacrevaz’s method are very plausible and that the values found at 9 GHz by Wagner’s method are probably slightly underestimated. What we observe with both methods is that the variation of the real part of permittivity remains very small as a function of concentration for a LiBr mass concentration over 40%—concentrations used in absorption machines. By comparing the results of the real part of permittivity extracted by Lacrevaz’s method at 100 MHz and the static permittivity data taken from [32] (see Table 7), we confirm that the values obtained at 100 MHz are quite correct for mass concentrations up to 33%.

As regards the imaginary part of the permittivity, we can see that it varies strongly with LiBr concentration, with a reversal of the direction of variation for LiBr mass concentrations between 33 and 41%. This is in line with what was predicted in Figure 1 and in agreement with generic Equations (1) and (2): the decrease in conductivity for solutions with concentrations above 35% is reflected in the imaginary part of the permittivity.

From these results, we can deduce that it will be difficult to make a sensor based on the real part of the permittivity, but that variations in the imaginary part are sufficient to make a concentration sensor between a hundred MHz and a few GHz.

### 3.4. Comparison Between the Two Extraction Methods on the Imaginary Part of the Permittivity

**Figure 12:**
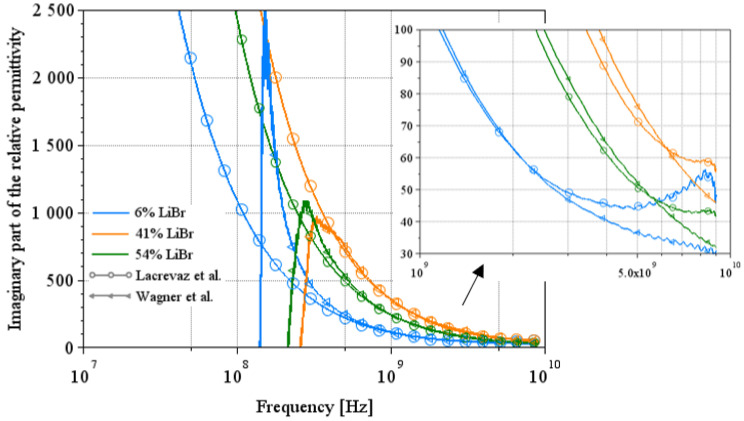
Comparison between the two extraction methods on the imaginary part of the complex permittivity for LiBr water solutions [7,12].

Figure 11 and Figure 12 show a comparison of the imaginary part of the permittivity extracted by the two methods on a few solution concentrations. Figure 11 shows the results for NaCl water solutions, and Figure 12 shows the results for LiBr water solutions over a wide spectrum from 10 MHz to 9 GHz.

In Figure 11, we can see a complementarity in their area of validity. Indeed, Lacrevaz’s method gives consistent results from low frequencies up to a few GHz; when results diverge from theory, Wagner’s method becomes consistent. The same behavior can be seen in the results for LiBr water solutions in Figure 12. A summary of the values of the imaginary part of the permittivity of LiBr water solutions is given in Table 8. The last line of this table shows the percentage change in the imaginary part of the permittivity for LiBr mass concentrations ranging from 41% to 54%. The imaginary part of the permittivity reaches a variation of 37% (calculated by dividing the difference between its two extreme values by the central value) over a wide frequency band: from 10 MHz to almost 9 GHz. The development of a sensor detecting variations in the imaginary part of permittivity (or the conductivity) at a particular frequency within this frequency range is, therefore, conceivable.

## 4. Conclusions

Using a low-cost SMA connector (a disposable probe if damaged by the corrosive solutions) and two permittivity extraction methods based on measurements that are fairly simple to implement, we were able to characterize low-conductivity and high-conductivity solutions up to 9 GHz. The measurements and extraction methods were first validated on ethanol and/or methanol, then tested on NaCl water solutions known from the literature, which enabled the limits of each extraction method to be determined. The technique was then applied to LiBr water solutions with LiBr mass concentrations ranging from 1% to 54%.

Because of high conductivity, extracting the real part of the permittivity remains difficult. It is underestimated in both permittivity extraction methods, but the tendency of the curves remains plausible. On the other hand, the imaginary part has consistent values with Lacrevaz’s method from low frequencies (10 MHz) up to a few GHz. Wagner’s method completes the spectrum of results above 5 GHz.

Compared to other works [32,33,34,35], we have added a frequency dimension to the study. In the hyperfrequency domain, it is possible to rely on the values of the imaginary part of the complex permittivity to obtain concentration information. These first results from microwave characterization of LiBr water solutions show that the conductivity of the solutions has a strong impact at least up to 9 GHz, and that the realization of a sensor would focus on detecting the imaginary part of the permittivity in the frequency range studied in this paper.

## Figures and Tables

**Table 1:** Parameters of reference liquids.

	Deionized Water	Ethanol 99.5%	Methanol 99.8%
*ε_m,S_*	87.85	28.56	37.90
*ε_m,∞_*	6.22	4.67	5.97
*α_S_* [10^−3^/°C]	−4.57	−6.34	−5.94
*α_∞_* [10^−3^/°C]	−5.79	−1.40	−2.76
*τ*_0_ [fs]	5.88	14.8	89.1
*E_A_* [kJ/mol]	18.02	23.11	15.72

**Table 2:** Conductivity measurements of reference solutions.

Reference Liquids	Measured Conductivity (µS/cm at 25 °C)
Deionized water	1
Ethanol	1.76
Methanol	2.2
Tap water	207

**Table 3:** Deionized water parameters based on [7,12]. The parameters are given for two temperatures close to room temperature.

	T = 20 °C		T = 25 °C	
	Ref. [12]	Ref. [7]	Ref. [12]	Ref. [7]
$\;\varepsilon_{S\_water}$	80.21	80.18	78.36	78.37
$\;\varepsilon_{\infty }$	5.6	5.5	5.2	5.38
$\tau_{water}\;$ (ps)	9.36	9.59	8.27	8.47

**Table 4:** Parameters of the NaCl water solutions as a function of concentration for two temperatures (20 °C and 25 °C) calculated using Equations (3)–(6).

	T = 20 °C				T = 25 °C			
NaCl Concentration	0.154 M	0.25 M	0.5 M	1 M	0.154 M	0.25 M	0.5 M	1 M
$\varepsilon_{S}$	78.07	76.75	73.54	68.14	76.22	74.91	71.74	66.40
*τ* (ps)	9.13	9.04	8.82	8.51	8.03	7.93	7.69	7.30
α (10^−2^)	1.07	1.61	2.59	2.63	1.02	1.53	2.43	2.31
$\sigma_{i}$ (S/m)	1.41	2.25	4.31	7.82	1.54	2.47	4.74	8.69

**Table 5:** Conductivity measurements of the NaCl water solutions (results are obtain for a 25 °C temperature).

	T = 25 °C			
NaCl Concentration	0.154 M	0.25 M	0.5 M	1 M
${\sigma_{i}}_{meas}\;$ (S/m)	1.42	2.22	4.14	7.63

**Table 6:** Concentration and conductivity measurements of LiBr water solutions.

Measured Concentration (% by Mass)	Correspondence in Moles/Liter (M)	Measured Conductivity (S/m at 25 °C)
1.2	0.14	1.11
6.4	0.77	4.59
12.8	1.62	8.08
23.8	3.28	12.63
32.8	4.89	14.56
41.0	6.61	14.38
47.8	8.25	12.52
53.9	9.94	9.90

**Table 7:** Comparison of the real part of the permittivity found on the Lacrevaz extraction curves at 100 MHz and the values taken from [32] using the Micheksen–Mollerup–Breil (MMB) model and their experimental correlation (EC).

		Real Part of the Permittivity		
LiBr Mass Concentration (%)	LiBr Concentration (M)	Lacrevaz’s Method [11] at 100 MHz	MMB Model in Ref. [32]	EC in [32]
6.4	0.77	68	66.4	65.5
12.8	1.62	56	55.5	52.5
23.8	3.28	37	40	36.4
32.8	4.89	25	31	26.4

**Table 8:** Results extracted by both methods to give an estimate of the imaginary part of permittivity as a function of frequency and as a function of LiBr mass concentration. In each box, the first line corresponds to a result from Wagner’s method and the second line to a result from Lacrevaz’s method; only plausible results are noted. The last line shows the percentage change in imaginary part of permittivity for LiBr mass concentrations ranging from 41% to 54%.

$\varepsilon_{r}^{\prime \prime }$							
LiBr Concentration	10 MHz	50 MHz	100 MHz	500 MHz	1 GHz	5 GHz	9 GHz
1%	2054	502	259	55	31 31	24	32
6%	8567	2091	1078	221	117 114	36	30
13%	15,245	3724	1920	391	205 198	51 56	36
24%	24,084	5898	3040	616	317 309	71 70	48
33%	27,676	6785	3498	708	359 353	78 74	50
41%	27,761	6804	3508	709	358 354	76 71	46
48%	23,826	5840	3011	609	306 304	65 62	40
54%	19,103	4676	2411	488	245 244	52 51	32
$\varepsilon_{r}^{\prime \prime }$ variation	37%	37%	37%	37%	37%	37%	36%

## Data Availability

The data that support the findings of this study are available from the corresponding author upon reasonable request.
